# Recent US bluetongue virus serotype 3 isolates found outside of Florida indicate evidence of reassortment with co-circulating endemic serotypes

**DOI:** 10.1099/jgv.0.000965

**Published:** 2017-11-09

**Authors:** Erin E. Schirtzinger, Dane C. Jasperson, Eileen N. Ostlund, Donna J. Johnson, William C. Wilson

**Affiliations:** ^1^​ United States Department of Agriculture, Agricultural Research Service, Arthropod-borne Animal Diseases Research Unit, 1515 College Avenue, Manhattan, KS 66502, USA; ^2^​ United States Department of Agriculture, Animal-Plant Health Inspection Service, National Veterinary Service Laboratories, Diagnostic Virology Laboratory, PO Box 844, Ames, IA 50010, USA

**Keywords:** bluetongue virus, serotype 3, orbivirus, reassortment, phylogenetics, United States

## Abstract

Since 1999, 11 serotypes of bluetongue virus (BTV) similar to Central American or Caribbean strains have been isolated in the southeastern United States, predominantly in Florida. The majority of the incursive serotypes have remained restricted to the southeastern US. In recent years, BTV serotype 3 (BTV-3) has been isolated in areas increasingly distant from Florida. The current study uses whole genome sequencing of recent and historical BTV-3 isolates from the US, Central America and the Caribbean with additional sequences from GenBank to conduct phylogenetic analyses. The individual segments of the BTV genome were analysed to determine if recent BTV-3 isolates are reassortants containing genomic segments from endemic US serotypes or if they retain a majority of Central American/Caribbean genotypes. The analyses indicate that BTV-3 isolates Mississippi 2006, Arkansas 2008 and Mississippi 2009 are closely related reassortants that contain five to six genomic segments that are of US origin and two to three segments of Central American/Caribbean origin. In contrast, the BTV-3 South Dakota 2012 isolate contains seven genomic segments that are more similar to isolates from Central American and the Caribbean. These different evolutionary histories of the BTV-3 isolates suggest that there are at least two different lineages of BTV-3 that are currently circulating in the US.

## Introduction

Bluetongue virus (BTV) is a non-enveloped, double-stranded RNA virus in the genus *Orbivirus*, family *Reoviridae.* The genome consists of ten segments that encode the seven structural (VP1-VP7) and four non-structural proteins (NS1, NS2, NS3/3a). The structural proteins are arranged in three layers comprising the outer capsid (VP2, VP5), the capsid (VP3, VP7) and the inner core (VP1, VP4, VP6) that surround the genomic RNA [1]. The non-structural proteins are responsible for cellular effects such as tubule and viral inclusion body formation (NS1 and NS2 respectively) and viral egress (NS3/3a) [1].

Bluetongue virus is transmitted by several species of biting midge of the genus *Culicoides* [2, 3]. BTV is the etiological agent of bluetongue disease (BTD), an economically important disease of domestic and wild ruminants. Impacts of BTD on the livestock industry are not limited to the production losses associated with the mortality/morbidity of BTD but also include international restrictions on the trade of animals from areas with BTD or specific BTV serotypes [4, 5]

BTD was first described in South Africa in the early 1900s [6, 7]. Initially, it was believed that BTV would emerge from Africa and devastate the world’s sheep population. However, as additional serotypes of BTV were identified on other continents without the presence of severe disease it was realized that BTV emergence was not a recent event [8–10]. Currently, at least 29 serotypes of BTV exist worldwide [11, 12]. In tropical and subtropical regions that support continuous vector populations and circulation of endemic BTV serotypes disease outbreaks are uncommon [10]. In these areas, outbreaks of disease are generally associated with the introduction of a new serotype often from a neighbouring region.

BTV was first isolated in the United States in the early 1950s, although BTD, known as ‘sore muzzle’, had been described earlier. By the early 1980s, four serotypes of BTV (10, 11, 13 and 17) were known to be endemic throughout the western and southern United States [8–10, 13]. BTV serotype 2 (BTV-2) was first detected in Florida in 1982 and has since become endemic in the southeastern United States [14, 15]. Only one presumably imported isolate of BTV-2 has been reported in California [16, 17]. In Central America and the Caribbean, serological typing of BTV isolates from the 1980s identified serotypes 1, 3, 4, 6, 8, 12 and 17 as endemic. More recently, sequencing of the serotype-specific segment of 1990s isolates from Central America and the Caribbean added six BTV serotypes (10, 11, 13, 14, 19 and 22) to this list [18]. *Culicoides insignis* is considered to be the primary vector of BTV in Central America and the Caribbean [2, 8, 10, 19]. *C. insignis* is also found throughout southern Florida, while *Culicoides sonorensis* is believed to be the primary vector of BTV in the rest of the United States. Data compiled from USDA, APHIS, National Veterinary Service Laboratories annual reports show that 11 invasive BTV serotypes were first isolated in the US between 1999 and 2015 [18, 20–39] (see Table S1, available in the online version of this article). Although, some of these isolations were from sick animals, many came from healthy animals being tested for export purposes. Nine of the 11 invasive serotypes were first isolated in Florida (3, 5, 6, 9, 14, 18, 19, 22, 24). Two additional serotypes, BTV-1, first isolated in 2004 in Louisiana [40] and BTV-12, first isolated in Texas in 2008 [32], were later isolated in Florida. While the US does not conduct active surveillance of circulating BTV serotypes in all areas, the available data suggests that Florida may be a common point of entry for invasive BTV serotypes. Many of the exotic serotypes continue to be sporadically isolated only in Florida, suggesting that either their persistence is due to the presence of a competent Florida vector or that the same serotypes are repeatedly introduced and then die out. In contrast, BTV-3 was first detected in Florida in 1999 and was repeatedly isolated over the next several years. However, since 2006 BTV-3 has been isolated in Mississippi, Arkansas, South Dakota and most recently in Texas [31, 33, 34, 39].

One reason for this increase in distribution of BTV-3 may be due to reassortment. During co-infection of the same animal or cell by two genetically different viral strains or serotypes of a segmented virus can create novel combinations of genetic segments during packaging of progeny viruses. These new reassortant viruses may display different phenotypic characteristics than either parental virus [41–43]. These new combinations of genetic elements can result in an increase or decrease in pathogenicity or transmissibility, in the ability to infect a new host or vector species, or in the persistence of an exotic strain [43].

This study uses whole genome sequencing and phylogenetic analyses of BTV isolates from the US, Central America and the Caribbean to investigate the hypothesis that BTV-3 has reassorted with co-circulating endemic strains enabling BTV-3 to utilize additional vectors with a larger geographic distribution and to extend from Florida into the Northern Plains.

## Results

Results from the phylogenetic analyses are presented as follows: the outer capsid [segments 2 (VP2) and 6 (VP5)], the capsid [segments 3 (VP3) and 7 (VP7)], the inner virus core [segments 1 (VP1), 4 (VP4) and 9 (VP6)] and non-structural proteins [segments 5 (NS1), 8 (NS2) and 10 (NS3/3a)]. The BTV-3 isolates Mississippi 2006, Arkansas 2008 and Mississippi 2009 (hereafter called the MAM clade) are found to form a distinct, well-supported clade in many of the phylogenetic trees.

### The outer capsid

**Fig. 1.**
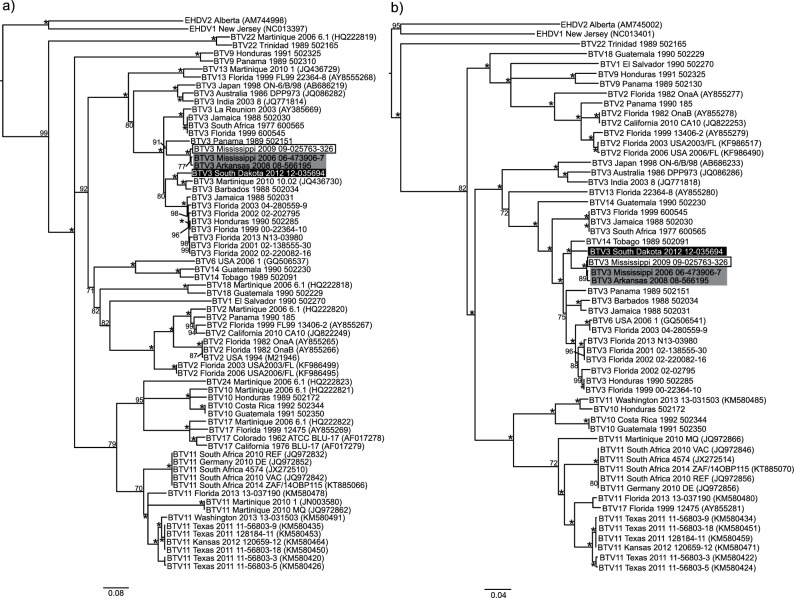
Phylogenetic trees of BTV genes encoding the outer capsid structural proteins. (a) Segment 2 (VP2). (b) Segment 6 (VP5). Trees were constructed in Geneious 8.0 using the nucleotide alignment, the neighbour-joining method, Jukes–Cantor distance and 1000 bootstrap pseudo-replicates. Bootstrap values are indicated either above or below the node. An asterisk indicates a bootstrap value of 100. BTV serotype 3 strains of interest are highlighted as follows: Mississippi 2009 (white box with black outline and black text), Mississippi 2006 and Arkansas 2008 (grey box with black text), and South Dakota 2012 (black box with white text).

The phylogenetic tree for segment 2, the determinant of serotype, shows sequences grouping according to serotype, as expected (Fig. 1a). All BTV-3 isolates form a single, well-supported clade with 84–86 % nucleotide identity (NI). The MAM clade groups with other BTV-3 sequences from Florida, Central America and the Caribbean, as well as South Dakota 2012. The nearest well-supported relative of the MAM clade for VP2 is BTV-3 Panama 1989. BTV-3 South Dakota 2012 is most closely related to sequences from BTV-3 Martinique 2010 and Barbados 1988 for segment 2 which forms the sister group to the BTV-3 Florida strains. For segment 6, the four isolates of interest cluster together with 95–96 % NI to BTV14 Tobago 1989 (Fig. 1b). In trees for both segments nearly all of the BTV-3 Florida isolates are found in a well-supported clade separate from the recent isolates. This suggests that for the isolates of interest, BTV-3 segments 2 and 6 are the descendants of a Central American/Caribbean BTV-3 ancestor.

### The capsid

**Fig. 2.**
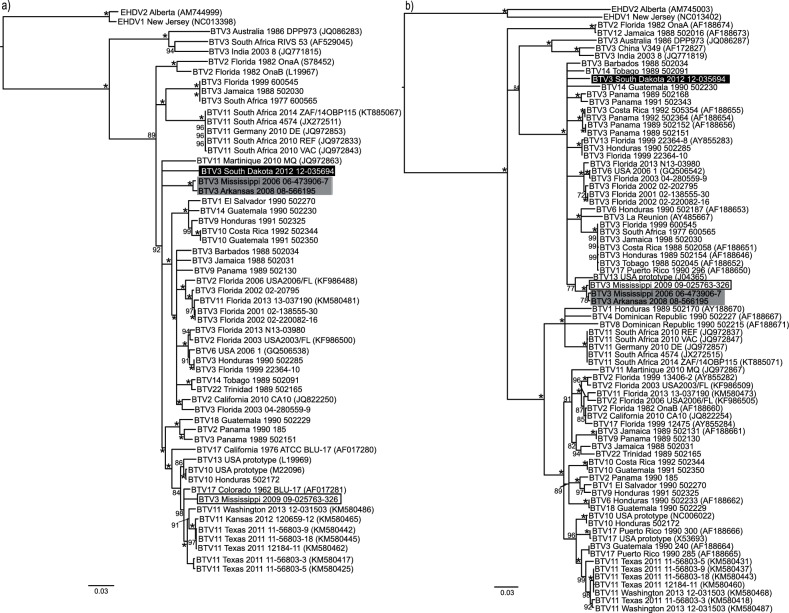
Phylogenetic trees of BTV genes encoding the capsid structural proteins. (a) Segment 3 (VP3). (b) Segment 7 (VP7). Trees were constructed in Geneious 8.0 using the nucleotide alignment, the neighbour-joining method, Jukes–Cantor distance and 1000 bootstrap pseudo-replicates. Bootstrap values are indicated either above or below the node. An asterisk indicates a bootstrap value of 100. BTV serotype 3 strains of interest are highlighted as follows: Mississippi 2009 (white box with black outline and black text), Mississippi 2006 and Arkansas 2008 (grey box with black text), and South Dakota 2012 (black box with white text).

The geographic origin of the segment 3 (VP3) sequences for the recent BTV-3 isolates is somewhat equivocal due to the majority of BTV-3 sequences grouping together (see Fig. 2a). Here, BTV-3 Mississippi 2006 and Arkansas 2008 form a well-supported clade separate from Mississippi 2009. These two isolates, along with BTV-3 South Dakota 2012 are part of a polytomy that includes nearly all of the US, Central American and Caribbean sequences. This suggests that more sequences are needed to determine the ancestry of segment 3 for Mississippi 2006, Arkansas 2008 and South Dakota 2012. Within this polytomy are two well-supported clades. One contains nearly all of the BTV-2 and BTV-3 Florida isolates with multiple serotypes from Central America and the Caribbean. The other includes multiple isolates of serotypes 10, 11, 13 and 17 from the western US with individual isolates of serotypes 2, 3 and 18 from Central America. BTV-3 Mississippi 2009 is included in this clade and shares 97–98 % NI with BTV-17 Colorado 1962 and BTV-11 isolates from Texas, Kansas and Washington 2011–2013. Analysis of segment 7, Fig. 2b, shows that the majority of BTV-3 isolates cluster together in a large polytomy with several well-supported clades. One of these contains the MAM clade and BTV-13 US prototype (96 % NI). The closest relative of BTV-3 South Dakota 2012 within this large polytomy is again undetermined. Increased numbers of sequences for segment 7 may aid in the resolution of these relationships. From the presence of large polytomies in the phylogenetic analyses of both segments, only two determinations can be made: BTV-3 Mississippi 2009 segment 3 is of US endemic origin and the MAM clade and South Dakota 2012 segment 7 sequences are of Central American/Caribbean origin.

### Inner virus core

**Fig. 3.**
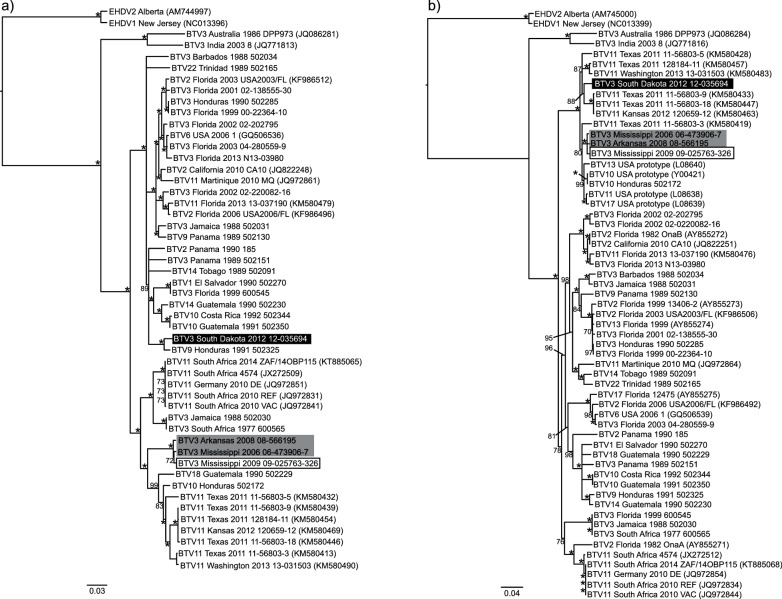

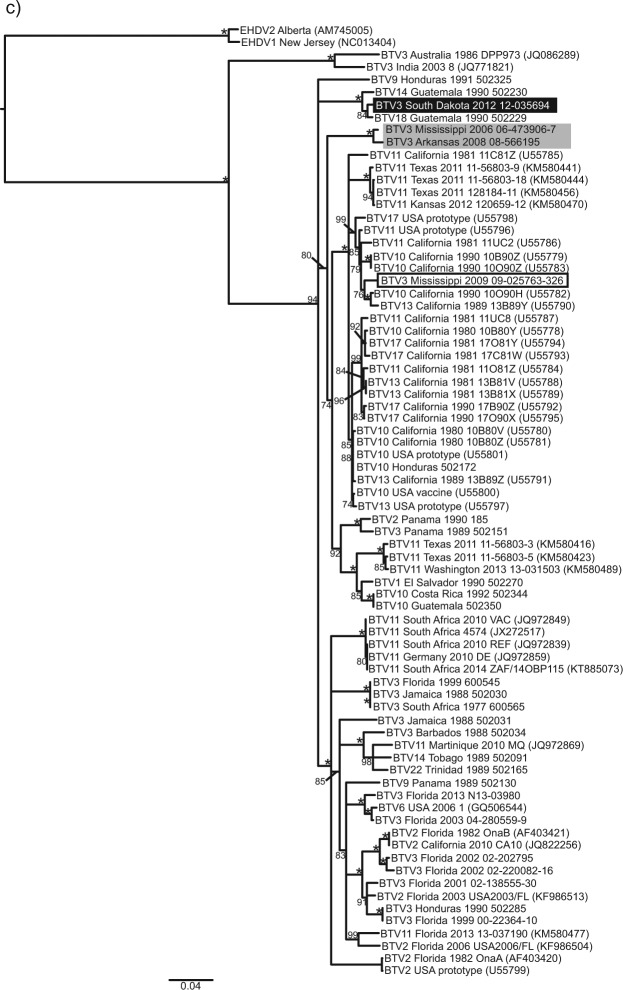
Phylogenetic trees of BTV genes encoding the inner virus core proteins. (a) Segment 1 (VP1). (b) Segment 4 (VP4). (c) Segment 9 (VP6). Trees were constructed in Geneious 8.0 using the nucleotide alignment, the neighbour-joining method, Jukes–Cantor distance and 1000 bootstrap pseudo-replicates. Bootstrap values are indicated either above or below the node. An asterisk indicates a bootstrap value of 100. BTV serotype 3 strains of interest are highlighted as follows: Mississippi 2009 (white box with black outline and black text), Mississippi 2006 and Arkansas 2008 (grey box with black text), and South Dakota 2012 (black box with white text).

The phylogenetic tree for segment 1 shows the MAM clade forming a well-supported sister group to a group of BTV-11 sequences from Texas, Kansas and Washington from 2011 to 2013 and two Central American isolates, BTV-18 Guatemala 1990 and BTV-10 from Honduras (91–93 % NI) (Fig. 3a). This grouping of BTV-3 and BTV-11 is further related to BTV-11 and BTV-3 from South Africa (91 % NI). BTV-3 South Dakota 2012, in contrast, is embedded within a group of multiple serotypes from Central America 1988–2013 (93–94 % NI) and is most closely related (98 % NI) to BTV-9 Honduras 1991. Analysis of segment 4 sequences shows the MAM clade belonging to a clade of US prototype strains for serotypes 10, 13, 11 and 17, BTV-10 from Honduras and a 2011 BTV-11 from Texas (Fig. 3b). BTV-3 South Dakota 2012 is also found to be closely related to isolates of BTV-11 Texas and Kansas 2011–2012 (97 % NI). Analysis of segment 9 sequences places BTV-3 Mississippi 2006 and Arkansas 2008 as the sister group to a large clade of western US isolates of serotypes 10, 11, 13 and 17 (see Fig. 3c). BTV-3 Mississippi 2009 is found within this large clade and is most closely related (97–98 % NI) to US BTV-10 and BTV-13 isolates from California 1989–90. BTV-3 South Dakota 2012 is most closely related to BTV-18 Guatemala (99 % NI) and BTV-14 Guatemala (98 % NI) for segment 9.

### Non-structural proteins

**Fig. 4.**
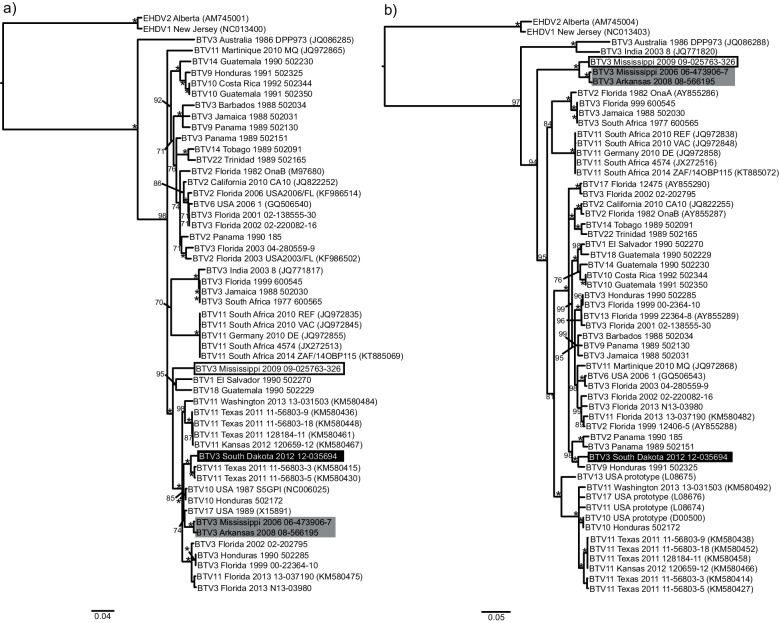

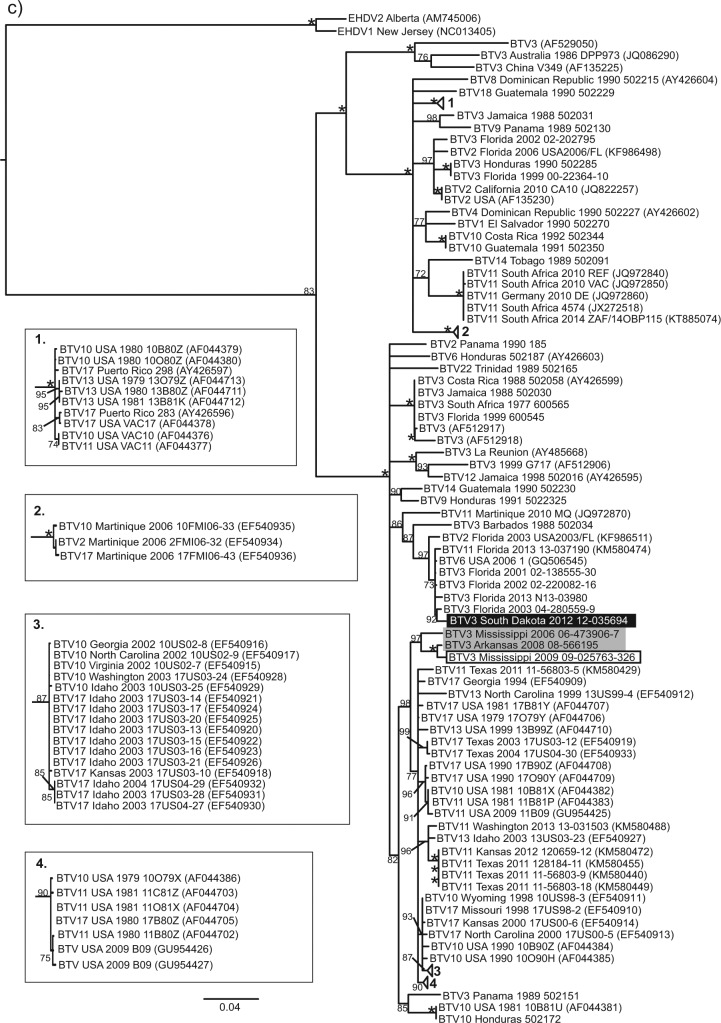
Phylogenetic trees of BTV genes encoding the non-structural proteins. (a) Segment 5 (NS1). (b) Segment 8 (NS2). (c) Segment 10 (NS3). Trees were constructed in Geneious 8.0 using the nucleotide alignment, the neighbour-joining method, Jukes–Cantor distance and 1000 bootstrap pseudo-replicates. Bootstrap values are indicated either above or below the node. An asterisk indicates a bootstrap value of 100. BTV serotype 3 strains of interest are highlighted as follows: Mississippi 2009 (white box with black outline and black text), Mississippi 2006 and Arkansas 2008 (grey box with black text), and South Dakota 2012 (black box with white text).

The non-structural proteins are encoded by segments 5 (NS1), 8 (NS2) and 10 (NS3/3A). In the phylogenetic tree for segment 5, (Fig. 4a), the four isolates of interest are all found within the same large, well-supported clade that includes US BTV-10, 11, 17, four isolates from Central America and several BTV-3 Florida isolates. BTV-3 Mississippi 2006 and Arkansas 2008, as well as BTV-3 South Dakota 2012 are located within the same clade that consists of isolates of BTV-11 Texas 2011, BTV-10 USA and Honduras and BTV-17 USA. Within this clade, BTV-3 Mississippi 2006 and Arkansas 2008 are most closely related to BTV-17 US 1989 with 98 % NI, while BTV-3 South Dakota 2012 shares a close relationship with BTV-11 Texas 2011 (98 % NI). Although BTV-3 Mississippi 2009 is also included in the same larger group of sequences, it is part of a polytomy and its closest relative is undetermined. The phylogeny for segment 8, shown in Fig. 4b, identifies the MAM clade as the well-supported sister group to all other BTV strains in the study except those from Australia and India. The placement of this clade suggests that the most recent ancestor for these isolates has not been sequenced. BTV-3 South Dakota 2012, however, shows a close relationship to sequences from Florida, Central America and the Caribbean with the sequence of BTV-9 Honduras 1991 being its closest relative (97 % NI). The segment 10 analysis places the MAM clade as the sister group to a large clade of US BTV-10, 11, 13, 17 isolates with 95–97 % NI (Fig. 4c). BTV-3 South Dakota 2012, in contrast, falls within a clade consisting of BTV-3, BTV-11 and BTV-6 isolates from Florida dating from 2001 to 2013. In summary, BTV-3 Mississippi 2006 and Arkansas 2008 have segments 5 and 10 that are of US endemic origin. Determination of US endemic ancestry for BTV-3 Mississippi 2009 can only be made for segment 10. BTV-3 South Dakota 2012 shows a mixture of ancestry for these segments with a US endemic origin for segment 5 and Central American/Caribbean origin for segments 8 and 10.

## Discussion

The current study uses whole genome sequencing and phylogenetic analyses of recent and historical isolates from the US, Central America and the Caribbean to determine if recent US BTV-3 isolates are reassortants with endemic US serotypes or if they retain a Central American/Caribbean signature. The analyses show that BTV-3 isolates from Mississippi 2006, Arkansas 2008 and Mississippi 2009 have very similar evolutionary histories that have resulted in the acquisition of a majority of genomic segments of US endemic serotype origin. In contrast, the BTV-3 South Dakota 2012 isolate has a majority of genomic segments that are more similar to BTV-3 sequences from Florida, Central America and the Caribbean. These different histories of the BTV-3 isolates suggest that there are at least two different lineages of reassortant BTV-3 currently circulating in the US (see Table 1).

**Table 1.** Geographic origin of each genomic segment for recent BTV-3 isolates as indicated by the phylogenetic analyses

**BTV segment**	**BTV protein**	**BTV3 Mississippi 2006**	**BTV3 Arkansas 2008**	**BTV3 Mississippi 2009**	**BTV3 South Dakota 2012**
2	VP2	CA/C*	CA/C	CA/C	CA/C
6	VP5	CA/C	CA/C	CA/C	CA/C
3	VP3	UND†	UND	US	UND
7	VP7	CA/C	CA/C	UND	CA/C
1	VP1	US‡	US	US	CA/C
4	VP4	US	US	US	US
9	VP6	US	US	US	CA/C
5	NS1	US	US	UND	US
8	NS2	UND	UND	UND	CA/C
10	NS3/3A	US	US	US	CA/C

*Central American/Caribbean.

†Undetermined.

‡United States.

Early studies of BTV isolates used electropherotypes or the pattern of RNA segments run on a polyacrylamide gel to show that significant variation existed between isolates of the same and different serotypes, as well as within isolates from the same area, outbreak or animal [44, 45]. Oligonucleotide fingerprinting of BTV RNA from US prototype isolates demonstrated that similarities between segment fragments from different serotypes were the result of reassortment in the field [46, 47]. Further research on field isolates of BTV has shown that reassortment is common where multiple serotypes circulate and/or live attenuated vaccines have been used [47, 48].

Reassortment of segmented viruses in cell culture has been shown to be essentially at random when the parental viruses have equal fitness [43, 49]. In studies where viruses were not matched for fitness, one constellation of viral segments became dominant with a small number of combinations being found at low frequencies [42, 43, 50]. Often when two serotypes were co-infected, only one serotype was found among the progeny [41, 42, 50, 51]. These data suggest that while all segments may be able to reassort, selection for fitness in the animal or vector will determine which reassortants are passed on [43, 52, 53]. In the current study, the BTV-3 Mississippi and Arkansas isolates only retained the BTV-3 segment that confers serotype, segment 2. In the field, previous exposure of livestock to endemic serotypes induces a protective response against subsequent exposures to these serotypes. Since serotype 3 is novel in the US and livestock outside of Florida are naïve, the segments that confer serotype may be selected over locally circulating endemic serotypes and may become dominant in the viral progeny.

Segments that code for non-structural proteins tend to be conserved due to negative selection that may be linked to functional constraint of these proteins. However, slight differences in fitness may lead to selection of specific segments. Ramig *et al.* [50] suggest that reassortant progeny viruses from co-infection of cell cultures contain more segments from the parental virus that was infected at a higher multiplicity of infection. This previous study, however, analysed reassortants by electropherotype. By repeating this former study using current sequencing methods, it may become possible to identify the sequence of parent virus infections in reassortant viruses. In the current study, the differences in evolutionary histories of the BTV-3 Mississippi and Arkansas isolates and the South Dakota isolate may reveal that in South Dakota only BTV-3 was circulating in the affected animals while multiple serotypes were circulating further south. Future studies using the BTV reverse genetics system and sequences from recent BTV-3 and endemic isolates may allow us to tease apart the influence of the different segments on virulence, replication and transmissibility in animals and vectors.

Unfortunately, due to a lack of continual surveillance of BTV serotypes throughout the US, isolations are only made during an outbreak of BTD or when testing for export purposes and usually only serotype is determined at the time. This does not allow us to determine with any precision where and when a virus has undergone reassortment or which parental strains were involved. Whole genome sequencing of new BTV-3 isolates is needed in order to continue tracking its evolution and persistence. Monitoring serotypes circulating in vectors, livestock and wild ruminants in areas where BTV-3 has previously been isolated will provide the information necessary to determine if recent isolates are the result of transient incursions from the southeastern US or if BTV-3 will become the next North American endemic serotype.

## Methods

A total of 27 bluetongue isolates of multiple serotypes (17 from BTV-3) from the United States, Central America and the Caribbean were sequenced for this study (see Table 2). BTVs not isolated in the United States were obtained from the Inter-American Bluetongue Project and the Onderstepoort Veterinary Institute virus library. Isolates from the United States were obtained from the National Veterinary Services Laboratories or the Arthropod-borne Animal Disease Research Unit reference collection. Viruses were typically isolated in embryonated chicken eggs or cattle pulmonary artery endothelial (CPAE) cells (ATCC CCL-209), followed by one to as many as seven passages in baby hamster kidney (BHK-21) (ATCC CCL-10) or CPAE cells. Total RNA was extracted from cells as previously described [54]. Viral double-stranded RNA was then purified by lithium chloride differential precipitation as described in [55] and subjected to whole genome sequencing using the sequence-independent amplification procedure described by [56] with modifications as described previously [57].

**Table 2.** Newly sequenced BTV isolates included in the current study

**Serotype**	**Isolate**	**Year**	**Location**	**Segment 1**	**Segment 2**	**Segment 3**	**Segment 4**	**Segment 5**	**Segment 6**	**Segment 7**	**Segment 8**	**Segment 9**	**Segment 10**
1	502270	1990	El Salvador	KY091928	KY092170	KY092117	KY092090	KY092089	KY092036	KY092009	KY091982	KY091957	KY091901
2	185	1990	Panama	KY091929	KY092149	KY092118	KY092091	KY092066	KY092037	KY092010	KY091983	KY091958	KY091902
3	502034	1988	Barbados	KY091930	KY092153	KY092119	KY092092	KY092067	KY092040	KY092011	KY091984	KY091959	KY091903
3	502285	1990	Honduras	KY091931	KY092154	KY092120	KY092093	KY092068	KY092041	KY092012	KY091985	KY091960	KY091904
3	502030	1988	Jamaica	KY091932	KY092155	KY092121	KY092094	KY092063	KY092042	KY092013	KY091986	KY091961	KY091905
3	502031	1988	Jamaica	KY091933	KY092156	KY092122	KY092095	KY092069	KY092043	KY092014	KY091987	KY091962	KY091906
3	N13-03980	2013	Florida	KY091945	KY092168	KY092134	KY092107	KY092079	KY092055	KY092026	KY091999	KY091956	KY091918
3	502151	1989	Panama	KY091934	KY092157	KY092123	KY092096	KY092086	KY092044	KY092015	KY091988	KY091963	KY091907
3	600565	1977	South Africa	KY091935	KY092158	KY092124	KY092097	KY092064	KY092045	KY092016	KY091989	KY091964	KY091908
3	00-22364-10	1999	Florida	KY091936	KY092159	KY092125	KY092098	KY092070	KY092046	KY092017	KY091990	KY091965	KY091909
3	02-138555-30	2001	Florida	KY091937	KY092160	KY092126	KY092099	KY092071	KY092047	KY092018	KY091991	KY091966	KY091910
3	02–202795	2002	Florida	KY091938	KY092161	KY092127	KY092100	KY092072	KY092048	KY092019	KY091992	KY091967	KY091911
3	02-220082-16	2002	Florida	KY091939	KY092162	KY092128	KY092101	KY092073	KY092049	KY092020	KY091993	KY091968	KY091912
3	04-280559-9	2003	Florida	KY091940	KY092163	KY092129	KY092102	KY092074	KY092050	KY092021	KY091994	KY091955	KY091913
3	06-473906-7	2006	Mississippi	KY091941	KY092164	KY092130	KY092103	KY092075	KY092051	KY092022	KY091995	KY091969	KY091914
3	08–566195	2008	Arkansas	KY091942	KY092165	KY092131	KY092104	KY092076	KY092052	KY092023	KY091996	KY091970	KY091915
3	09-025763-326	2009	Mississippi	KY091943	KY092166	KY092132	KY092105	KY092077	KY092053	KY092024	KY091997	KY091971	KY091916
3	12–035694	2012	South Dakota	KY091944	KY092167	KY092133	KY092106	KY092078	KY092054	KY092025	KY091998	KY091972	KY091917
3	600545	1999	Florida	KY091946	KY092169	KY092135	KY092108	KY092065	KY092056	KY092027	KY092000	KY091973	KY091919
9	502325	1991	Honduras	KY091947	KY092145	KY092136	KY092109	KY092080	KY092038	KY092028	KY092001	KY091974	KY091920
9	502130	1989	Panama	KY091948	KY092146	KY092137	KY092110	KY092081	KY092039	KY092029	KY092002	KY091975	KY091921
10	502344	1992	Costa Rica	KY091949	KY092150	KY092138	KY092111	KY092082	KY092060	KY092030	KY092003	KY091976	KY091922
10	502350	1991	Guatemala	KY091950	KY092151	KY092139	KY092112	KY092083	KY092061	KY092031	KY092004	KY091977	KY091923
14	502230	1990	Guatemala	KY091951	KY092147	KY092140	KY092113	KY092084	KY092057	KY092032	KY092005	KY091978	KY091924
14	502091	1989	Tobago	KY091952	KY092148	KY092141	KY092114	KY092085	KY092058	KY092033	KY092006	KY091979	KY091925
18	502229	1990	Guatemala	KY091953	KY092152	KY092142	KY092115	KY092087	KY092059	KY092034	KY092007	KY091980	KY091926
22	502165	1989	Trinidad	KY091954	KY092144	KY092143	KY092116	KY092088	KY092062	KY092035	KY092008	KY091981	KY091927

Library preparation and sequencing was performed using the Ion Torrent OneTouch ES, Personal Genome Machine with the Ion Xpress Plus fragmentation library kit, Xpress barcode adapters, Ion library quantitation kit, OneTouch 200 template kit v2, Ion PGM sequencing 400 kit and Ion 314 chip following the protocols recommended by the manufacturer (Life Technologies, Grand Island, NY). Briefly, approximately 1 µg of viral cDNA was fragmented, barcoded and quantitated. Template generation, enrichment and sequencing were performed on the appropriate Ion OneTouch instruments (Life Technologies, Grand Island, NY).

Standard Flow-gram Format files were imported into Geneious 6.0 (Biomatters) for contig creation. Partial contigs were assembled and blasted against the NCBI nucleotide database to determine reference sequences that were then used for reference-based assemblies.

### Phylogenetic analysis

In order to encompass the currently known diversity of isolates from the United States, Central America and Caribbean regions additional BTV segment sequences were downloaded from GenBank. The genome segments from serotypes 1 and 2 of the closely related orbivirus, epizootic haemorrhagic disease virus (EHDV) were used to root the trees. The newly sequenced BTV genomes have been deposited in GenBank, KY091901-KY092170 (Table 2). GenBank accession numbers for all viral segments included in the study are located in Table S2.

Alignments of each BTV segment were made in Geneious 8.0 (www.geneious.com, [58]) using the MAFFT alignment option with default values. The ORF for each segment was determined using the Find ORFs option and the UTRs at the 5′ and 3′ ends were removed.

Phylogenetic trees for each segment were estimated by the neighbour-joining method under the Jukes–Cantor distance model in Geneious 8.0. The homologous segments of EHDV-1 and EHDV-2 were used as outgroups to root the trees. Support values for nodes were estimated by 1000 bootstrap pseudo-replicates. Nodes with bootstrap values less than 70 were considered to be unsupported. Four BTV-3 strains, Mississippi 2006, Arkansas 2008, Mississippi 2009 and South Dakota 2012 were evaluated for potential reassortment events by comparison of nearest neighbours in phylogenetic trees for each individual segment. Reassortment can be demonstrated by the degree of topological congruence between phylogenetic trees of individual genome segments. When all of the segments for a viral strain share the same ancestor–descendent relationships the topologies of the phylogenetic trees of each segment will be very similar or identical. A segment from a co-infecting strain will have different ancestor–descendent relationships and therefore the closest relatives will differ in the phylogenetic tree for that segment.

## Supplementary Data

72 Supplementary File 1
