# Supplementary material for: Recent US bluetongue virus serotype 3 isolates found outside of Florida indicate evidence of reassortment with co-circulating endemic serotypes
Source: J Gen Virol. 2017 Nov 9;99(2):157–68. doi: 10.1099/jgv.0.000965 (PMC5882081; doi:10.1099/jgv.0.000965)
Supplement: Supplementary File 1 [file jgv-99-157-s001.pdf]

Supplementary Table 1. Bluetongue serotype, species affected and location of virus isolations and PCR positive samples typed at the National Veterinary Service Laboratories from 1995-2015.

Year	Serotypes	Species	Location
2015	3, **6**, 10, 13, 17, **19**, **22**, **24**	Bighorn sheep, deer, sheep, cattle, yak	AZ, CA, FL, ID, NV, OK, TX, WA
2014	10, 11, 17, ***18***	Bighorn sheep, deer, alpaca, cattle, goat	CO, FL, ID, MO, NE, NJ
2013	**1**, **2**, **3**, 10, 11, 13, 17	Cattle, pronghorn, goat, deer, bison, sheep, llama, alpaca	CA, FL, IN, IA, MO, NE, OH, OK, PA, SD, TX, WA
2012	3, **9**, 10, 11, **12**, 13, 17	Deer, elk, cattle, dog, sheep, goat, bighorn sheep	AZ, CA, FL, IL, IA, KS, LA, NE, NM, ND, OK, SD, TX
2011	11, **12**, 13, 17	Cattle, deer, sheep, pronghorn	CA, KY, MO, NC, OK, PA, TX, WY
2010	1, 10, 11, **12**	Cattle, sheep, deer	CA, FL
2009	3, 11, 14	Deer	MS, OK, TX
2008	3, ***9***, *12*, 17	Cattle, sheep, deer	AR, CA, FL, KS, OK, TX
2007	11, 17, ***24***	Cattle, deer, sheep, pronghorn	CA, FL, IA, MO, MT, NM, SC, SD
2006	**2**, 3, ***6***, 11, 17	Cattle, sheep, deer	FL, IA, IL, KS, KY, MO, MS, NE,NM,OR, SD, TX, WA
2005	11, **22**	Bighorn sheep, cattle, deer	CA, IL, MD, SD, NE, OH
2004	*1*, 17	Cattle, deer, sheep	AL, CO, FL, LA, MT, NE, TX
2003	2, **3**, ***5***, ***14***, ***19***	Alpaca, bighorn sheep, cattle, deer, sheep	CA, CO, FL, IL, OK, TX
2002	**3**, 10, 17, ***22***	Cattle, deer, sheep	AZ, CA, FL, KS, NM, SC, TX
2001	**3**, 13, 17	Bighorn sheep, cattle, deer, elk, goats, sheep	AZ, CA, FL, KS, MO, NM, SD, TX
2000	11, 13, 17	Cattle, sheep	CA, KS, MD, MT, NE, NM, OR
1999	2, ***3***, 10, 11, 13, 17	Bighorn sheep, cattle, sheep	CA, FL, ID, LA, OK, WA
1998	10, 11, 17	Bighorn sheep, cattle, deer, gerenuk, sheep	AZ, CA, FL, ID, KS, NV, NM, WA
1997	11, 13	Bighorn sheep, cattle, deer, gerenuk, sheep	AZ, CA, FL, NM
1996	11, 13, 17	Bighorn sheep, bongo, cattle, deer, sheep	AZ, CA, FL, LA, NM, OR, TX
1995	11	Cattle	ID

*Summarized from annual reports to the Bluetongue and Bovine Retrovirus Committee published in the Proceeding of the Annual Meeting of the United States Animal Health Association 1996-2015.
Bold – indicates serotypes found in Florida.
Italics – indicates first identification of serotype

Supplementary Table 2: GenBank numbers of all sequences included in the current study

				BTV Segment									
Serotype	Location	Date	Isolate	L1	L2	L3	M4	M5	M6	S7	S8	S9	S10
1	El Salvador	1990	502270	KY091928	KY092170	KY092117	KY092090	KY092089	KY092036	KY092009	KY091982	KY091957	KY091901
2	California	2010	CA10	JQ822248	JQ822249	JQ822250	JQ822251	JQ822252	JQ822253	JQ822254	JQ822255	JQ822256	JQ822257
2	Florida	2006	USA2006/FL	KF986496	KF986495	KF986488	KF986492	KF986514	KF986490	KF986505	KF986508	KF986504	KF986498
2	Florida	2003	USA2003/FL	KF986512	KF986499	KF986500	KF986506	KF986502	KF986517	KF986509	KF986510	KF986513	KF986511
2	Florida	1982	OnaA		AY855265		AY855271		AY855277	AF188674	AY855286		
2	Florida	1999	13406-2		AY855267		AY855273		AY855279	AY855282	AY855288		
2	Florida	1982	OnaB		AY855266		AY855272		AY855278	AF188660	AY855287		
2	Martinique	2006	6.1		HQ222820								
2	Martinique	2006	2FMI06-32										EF540934
2	Panama	1990	185	KY091929	KY092149	KY092118	KY092091	KY092066	KY092037	KY092010	KY091983	KY091958	KY091902
2	USA	1994	US		M21946								
2	USA	1982	OnaB			L19967		M97680					
2	USA	1982	OnaA			S78452							
2	USA		prototype									U55799	
2	USA												AF135230
3	Arkansas	2008	08-566195	KY091942	KY092165	KY092131	KY092104	KY092076	KY092052	KY092023	KY091996	KY091970	KY091915
3	Australia	1986	DPP973	JQ086281	JQ086282	JQ086283	JQ086284	JQ086285	JQ086286	JQ086287	JQ086288	JQ086289	JQ086290
3	Barbados	1988	502034	KY091930	KY092153	KY092119	KY092092	KY092067	KY092040	KY092011	KY091984	KY091959	KY091903
3	China		V349							AF172827			AF135225
3	Costa Rica	1988	502058										AY426599
3	Florida	2013	N13-03980	KY091945	KY092168	KY092134	KY092107	KY092079	KY092055	KY092026	KY091999	KY091956	KY091918
3	Florida	1999	00-22364-10	KY091936	KY092159	KY092125	KY092098	KY092070	KY092046	KY092017	KY091990	KY091965	KY091909
3	Florida	2001	02-138555-30	KY091937	KY092160	KY092126	KY092099	KY092071	KY092047	KY092018	KY091991	KY091966	KY091910
3	Florida	2002	02-202795	KY091938	KY092161	KY092127	KY092100	KY092072	KY092048	KY092019	KY091992	KY091967	KY091911
3	Florida	2002	02-220082-16	KY091939	KY092162	KY092128	KY092101	KY092073	KY092049	KY092020	KY091993	KY091968	KY091912
3	Florida	2003	04-280559-9	KY091940	KY092163	KY092129	KY092102	KY092074	KY092050	KY092021	KY091994	KY091955	KY091913
3	Florida	1999	600545	KY091946	KY092169	KY092135	KY092108	KY092065	KY092056	KY092027	KY092000	KY091973	KY091919
3	Guatemala	1990	240							AF188664			
3	Honduras	1990	502285	KY091931	KY092154	KY092120	KY092093	KY092068	KY092041	KY092012	KY091985	KY091960	KY091904
3	India	2003	8	JQ771813	JQ771814	JQ771815	JQ771816	JQ771817	JQ771818	JQ771819	JQ771820	JQ771821	JQ771822
3	Jamaica	1988	502030	KY091932	KY092155	KY092121	KY092094	KY092063	KY092042	KY092013	KY091986	KY091961	KY091905
3	Jamaica	1988	502031	KY091933	KY092156	KY092122	KY092095	KY092069	KY092043	KY092014	KY091987	KY091962	KY091906
3	Japan	1998	ON-6/B/98		AB686219								
3	La Reunion	2003			AY485669								AY485668
3	Martinique	2010	10.02		JQ436730								
3	Mississippi	2006	06-473906-7	KY091941	KY092164	KY092130	KY092103	KY092075	KY092051	KY092022	KY091995	KY091969	KY091914
3	Mississippi	2009	09-025763-326	KY091943	KY092166	KY092132	KY092105	KY092077	KY092053	KY092024	KY091997	KY091971	KY091916
3	Panama	1989	502151	KY091934	KY092157	KY092123	KY092096	KY092086	KY092044	KY092015	KY091988	KY091963	KY091907
3	South Africa	1977	600565	KY091935	KY092158	KY092124	KY092097	KY092064	KY092045	KY092016	KY091989	KY091964	KY091908
3	South Dakota	2012	12-035694	KY091944	KY092167	KY092133	KY092106	KY092078	KY092054	KY092025	KY091998	KY091972	KY091917
3													AF529050
3													AF512917
3													AF512918
3			G717										AF512906
4	Dominican Republic	1990	502227							AF188667			AY426602
6	Honduras	1990	502233							AF188662			
6	Honduras		502187										AY426603
6	USA	2006	1	GQ506536	GQ506537	GQ506538	GQ506539	GQ506540	GQ506541	GQ506542	GQ506543	GQ506544	GQ506545
8	Dominican Republic	1990	502215							AF188671			AY426604
9	Honduras	1991	502325	KY091947	KY092145	KY092136	KY092109	KY092080	KY092038	KY092028	KY092001	KY091974	KY091920
9	Panama	1989	502130	KY091948	KY092146	KY092137	KY092110	KY092081	KY092039	KY092029	KY092002	KY091975	KY091921
10	California	1990	10O90H									U55782	
10	California	1990	10B90Z									U55779	
10	California	1990	10O90Z									U55783	
10	California	1980	10B80Y									U55778	

10	California	1980	10O80V									U55780	
10	California	1980	10O80Z									U55781	
10	Costa Rica	1992	502344	KY091949	KY092150	KY092138	KY092111	KY092082	KY092060	KY092030	KY092003	KY091976	KY091922
10	Georgia	2002	10US02-8										EF540916
10	Guatemala	1991	502350	KY091950	KY092151	KY092139	KY092112	KY092083	KY092061	KY092031	KY092004	KY091977	KY091923
10	Honduras	1989	502170							AF188670			
10	Idaho	2003	10US03-25										EF540929
10	Martinique	2006	6.1		HQ222821								
10	Martinique	2006	10FMI06-33										EF540935
10	North Carolina	2002	10US02-9										EF540917
10	USA		prototype			M22096	Y00421			NC006022	D00500	U55801	
10	USA		vaccine									U55800	
10	USA	1981	10B81X										AF044382
10	USA	1990	10O90Z										AF044384
10	USA	1990	10O90H										AF044385
10	USA	1981	10B81U										AF044381
10	USA	1979	10O79X										AF044386
10	USA	1980	10B80Z										AF044379
10	USA	1980	10O80Z										AF044380
10	USA		VAC10										AF044376
10	Virginia	2002	10US02-7										EF540915
10	Washington	2003	17US03-24										EF540928
10	Wyoming	1998	10US98-3										EF540911
11	California	1981	11C81Z									U55785	
11	California	1981	11UC2									U55786	
11	California	1981	11UC8									U55787	
11	California	1981	11O81Z									U55784	
11	Florida	2013	13-037190	KM580479	KM580478	KM580481	KM580476	KM580475	KM580480	KM580473	KM580482	KM580477	KM580474
11	Germany	2010	DE	JQ972851	JQ972852	JQ972853	JQ972854	JQ972855	JQ972856	JQ972857	JQ972858	JQ972859	JQ972860
11	Kansas	2012	120659-12	KM580469	KM580464	KM580465	KM580463	KM580467	KM580471	KM580468	KM580466	KM580470	KM580472
11	Martinique	2010	MQ	JQ972861	JQ972862	KQ972863	JQ972864	JQ972865	JQ972866	JQ972867	JQ972868	JQ972869	JQ972870
11	Martinique	2010	1		JN003580								
11	South Africa	2010	REF	JQ972831	JQ972832	JQ972833	JQ972834	JQ972835	JQ972836	JQ972837	JQ972838	JQ972839	JQ972840
11	South Africa	2010	VAC	JQ972841	JQ972842	JQ972843	JQ972844	JQ972845	JQ972846	JQ972847	JQ972848	JQ972849	JQ972850
11	South Africa		4574	JX272509	JX272510	JX272511	JX272512	JX272513	JX272514	JX272515	JX272516	JX272517	JX272518
11	South Africa	2014	ZAF/14OBP115	KT885065	KT885066	KT885067	KT885068	KT885069	KT885070	KT885071	KT885072	KT885073	KT885074
11	Texas	2011	11-56803-3	KM580413	KM580420	KM580417	KM580419	KM580415	KM580422	KM580418	KM580414	KM580416	KM580422
11	Texas	2011	11-56803-5	KM580432	KM580426	KM580425	KM580428	KM580430	KM580424	KM580431	KM580427	KM580423	KM580429
11	Texas	2011	11-56803-9	KM580439	KM580435	KM580442	KM580433	KM580436	KM580434	KM580437	KM580438	KM580441	KM580440
11	Texas	2011	11-56803-18	KM580446	KM580450	KM580445	KM580447	KM580448	KM580451	KM580443	KM580452	KM580444	KM580449
11	Texas	2011	128184-11	KM580454	KM580453	KM580262	KM580457	KM580461	KM580459	KM580460	KM580458	KM580456	KM580455
11	USA		prototype				L08638				L08674	U55796	
11	USA	1981	11B81P										AF044383
11	USA	2009	11B09										GU954425
11	USA	1981	11C81Z										AF044703
11	USA	1981	11O81X										AF044704
11	USA	1980	11B80Z										AF044702
11	USA		VAC11										AF044377
11	Washington	2013	13-031503	KM580490	KM580491	KM580486	KM580483	KM580484	KM580485	KM580487	KM580492	KM580489	KM580488
12	Jamaica	1988	502016							AF188673			AY426595
13	California	1989	13B89Y									U55790	
13	California	1981	13B81V									U55788	
13	California	1981	13B81X									U55789	
13	California	1989	13B89Z									U55791	
13	Florida	1999	22364-8		AY855268		AY855274		AY855280	AY855283	AY855289		
13	Idaho	2003	13US03-23										EF540927
13	Martinique	2010	1		JQ436729								
13	North Carolina	1999	13US99-4										EF540912

13	USA		prototype			L19969	L08640				L08676	U55797	
13	USA	1999	13B99Z										AF044710
13	USA	1979	13O79Z										AF044713
13	USA	1980	13B80Z										AF044711
13	USA	1981	13B81K										AF044712
14	Guatemala	1990	502230	KY091951	KY092147	KY092140	KY092113	KY092084	KY092057	KY092032	KY092005	KY091978	KY091924
14	Tobago	1989	502091	KY091952	KY092148	KY092141	KY092114	KY092085	KY092058	KY092033	KY092006	KY091979	KY091925
17	California	1976	BLU-17 198		AF017279	AF017280							
17	California	1981	17O81Y									U55794	
17	California	1981	17C81W									U55793	
17	California	1990	17B90Z									U55792	
17	California	1990	17O90X									U55795	
17	Colorado	1962	ATCC BLU-17		AF017278	AF017281							
17	Florida	1999	12475		AY855269		AY855275		AY855281	AY855284	AY855290		
17	Georgia	1991											EF540909
17	Idaho	2003	17US03-14										EF540921
17	Idaho	2003	17US03-14										EF540924
17	Idaho	2003	17US03-20										EF540925
17	Idaho	2003	17US03-13										EF540920
17	Idaho	2003	17US03-15										EF540922
17	Idaho	2003	17US03-16										EF540923
17	Idaho	2003	17US03-21										EF540926
17	Idaho	2004	17US04-29										EF540932
17	Idaho	2003	17US03-28										EF540931
17	Idaho	2003	17US04-27										EF540930
17	Kansas	2000	17US00-6										EF540914
17	Kansas	2003	17US03-10										EF540918
17	Martinique	2006	6.1		HQ222822								
17	Martinique	2006	17FMI06-43										EF540936
17	Missouri	1998	17US98-2										EF540910
17	North Carolina	2000	17US00-5										EF540913
17	Puerto Rico	1990	300							AF188666			
17	Puerto Rico	1990	285							AF188665			
17	Puerto Rico	1990	296							AF188650			
17	Puerto Rico		298										AY426597
17	Puerto Rico		283										AY426596
17	Texas	2003	17US03-12										EF540919
17	Texas	2004	17US04-30										EF540933
17	USA		prototype				L08639			X53693		U55798	
17	USA	1989						X15891					
17	USA	1981	17B81Y										AF044707
17	USA	1979	17O79Y										AF044706
17	USA	1990	17B90Z										AF044708
17	USA	1990	17O90Y										AF044709
17	USA	1980	17B80Z										AF044705
17	USA		VAC17										AF044378
18	Guatemala	1990	502229	KY091953	KY092152	KY092142	KY092115	KY092087	KY092059	KY092034	KY092007	KY091980	KY091926
18	Martinique	2006	6.1		HQ222818								
22	Martinique	2006	6.1		HQ222819								
22	Trinidad	1989	502165	KY091954	KY092144	KY092143	KY092116	KY092088	KY092062	KY092035	KY092008	KY091981	KY091927
24	Martinique	2006	6.1		HQ222823								
EHDV1			New Jersey	NC013396	NC013397	NC013398	NC013399	NC013400	NC013401	NC013402	NC013403	NC013404	NC103405
EHDV2			Alberta	AM744997	AM744998	AM744999	AM745000	AM745001	AM745002	AM745003	AM745004	AM745005	AM745006
	USA	2009	B09										GU954426
	USA	2009	B09										GU954427
